# CIRM tools and technologies: Breaking bottlenecks to the development of stem cell therapies

**DOI:** 10.1002/sctm.20-0055

**Published:** 2020-07-03

**Authors:** Lila R. Collins, Kelly A. Shepard

**Affiliations:** ^1^ California Institute for Regenerative Medicine Oakland California USA

**Keywords:** animal Models, cellular therapy, clinical translation, differentiation, direct cell conversion, induced pluripotent stem cells (iPSCs), stem cell transplantation

## Abstract

The California Institute for Regenerative Medicine (CIRM) has a mission to accelerate stem cell treatments to patients with unmet medical needs. This perspective describes successful examples of work funded by CIRM's New Cell Lines and Tools and Technologies Initiatives, which were developed to address bottlenecks to stem cell research and translation. The tools developed through these programs evolved from more discovery‐oriented technologies, such as disease models, differentiation processes, and assays, to more translation focused tools, including scalable good manufacturing processes, animal models, and tools for clinical cell delivery. These tools are available to the research community and many are facilitating translation of regenerative therapeutics today.

## Graphical abstract

Focused efforts to develop novel tools and technologies have advanced regenerative medicine from discovery to development. Key CIRM investments have included methods to scale up and manufacture stem cells and differentiated derivatives in xeno‐free conditions; novel disease and preclinical models; tools for safe and efficacious delivery of cell therapies; and new methods to engineer and deliver genes for therapeutic applications.

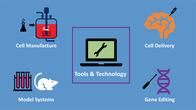

**Significance statement**

At California Institute for Regenerative Medicine's (CIRM) inception, the potential of human stem cells to alleviate disease was clear. However, the inaccessibility of relevant living human cells for study stymied the understanding of human disease. Clinically appropriate manufacturing methods, quality control tools, animal models to evaluate cell therapies, and delivery tools for living products were limiting. CIRM's comprehensive vision to build the stem cell therapy field by investing in the development of tools and technologies required to support this emerging discipline has resulted in the examples of successfully developed tools discussed in this perspective. These tools are helping to realize stem cell therapy's promise.

## 1 INTRODUCTION

Since its inception in 2004, the California Institute for Regenerative Medicine (CIRM) has funded the discovery and development of regenerative therapies for patients with unmet medical needs. Most of these treatments are stem cell‐derived therapies, a relatively new therapeutic modality that faces challenges in manufacturing of cells and viral vectors, genetic modification of cells, development of novel in vitro assays, preclinical modeling, and in vivo cell delivery. To facilitate the development of such therapies, CIRM issued two types of Requests for Applications (RFAs). The New Cell Lines (NCL) RFA addressed the lack of useful human embryonic stem cell (hESC) lines and the need for in vitro models of human diseases, and the Tools and Technologies (TNT) RFAs addressed technical challenges in manufacturing, disease modeling, and in vivo cell delivery. A core theme of TNT RFAs has been their multidisciplinary nature, recruiting the expertise of immunologists, biomedical and materials scientists, mechanical and process engineers to work with developmental and cell biologists, forming a stronger community of scientists to solve these knowledge and technological gaps. A total of 81 projects (17 NCL, 64 TNT) were funded in the amount of approximately $105M ($24M NCL, $81M TNT). Many of these tools are recently developed, and their ultimate impacts remain to be established. In this perspective, we will describe some examples of outcomes from these RFAs and elaborate on how tool‐focused discovery programs can both directly and indirectly advance the field.

## 2 IN VITRO DISEASE MODELS

The invention of induced pluripotent stem cells (iPSCs) coupled with directed differentiation protocols has enabled the study of previously inaccessible human cell types, including neurons, glia, and cardiomyocytes (CMs). By deriving these cell types in vitro from both patient and control iPSC, scientists are able to identify cellular and molecular differences that may account for or reflect disease mechanisms. This ability has opened the possibility of understanding the cellular and molecular basis of complex polygenic neurological diseases that have stymied medicine for decades. In addition, iPSC models can be used to develop cell‐based assays to screen for disease‐modifying drugs. Through the NCL and TNT RFAs, CIRM funded both model enabling tools, such as direct reprogramming methods^1^, ^2^ and the development of multiple successful “disease in a dish” models reflecting a variety of organ systems.^3^, ^4^, ^5^, ^6^, ^7^, ^8^, ^9^, ^10^, ^11^, ^12^, ^13^ The bulk of these targeted previously difficult to study neurological diseases, including psychiatric disorders,^3^ various autism spectrum disorders,^6^, ^7^, ^8^ aneuploidies,^14^ and genetic forms of neurodegeneration^5^, ^9^, ^10^ (Table 1). One such example is elaborated here.

**TABLE 1:** Tools and technologies for modeling human disease in vitro

PI/program	Disease or technology	Key contribution
Marius Wernig (TNT)	Single step reprogramming of hPSC to neurons	Rapid, derivation of excitatory forebrain neurons capable of in vitro and in vivo synapse formation. Model and cell therapy enabling.
Fred Gage (NCL)	Schizophrenia	Recapitulation of disease phenotype in vivo demonstrates that a genetically complex disease can be modeled in hiPSC.
Fred Gage (NCL)	Rett Syndrome	ASD model recapitulates features of Rett patient neurons, including decreased soma size and synaptic defects; enables study of potential therapeutic strategies.
Ricardo Dolmetsch and Theo Palmer (TNT)	Phelan McDermid Syndrome	ASD model demonstrates synaptic defect phenotype; enables study of potential therapeutic strategies.
Ricardo Dolmetsch and Theo Palmer (TNT)	Timothy syndrome	ASD model demonstrates neuronal calcium signaling and neurotransmitter defects; enables study of potential therapeutic strategies.
Larry Goldstein (TNT)	Alzheimer's disease	Methods for establishing and confirming quality of iPSC lines for Alzheimer's disease modeling; supported demonstration that a disease of aging can be modeled in reprogrammed cells.
Renee Reijo Pera (NCL)	Parkinson's disease	Alpha synuclein triplication is adequate to render patient neurons sensitive to oxidative stress induced cell death.
Leslie Thompson (NCL)	Huntington's disease	Model demonstrates CAG repeat based dose dependent sensitivity of HD neurons to multiple stressors, mirroring the repeat associated age of onset in the disease.
Fen‐Bao Gao and Robert Farese (NCL)	Frontotemporal Dementia	Progranulin insufficient neurons display increased sensitivity to cellular stress induced by inhibitors of the PI3K and MAPK pathway, which could be rescued with progranulin expression. Licensed to Bluefield Project to Cure Frontotemporal Dementia.
Richard Gatti (TNT)	Ataxia Telangiectasia	ATM patient neurons display mitochondrial and DNA damage repair defects that can be ameliorated by small molecule read through compounds.
Bruce Conklin (NCL, TNT)	LQTS	Model recapitulates features of LQTS, drug screening tool for personalized medicine. Available to both academic and commercial entities: Coriell Institute NIGMS iPSC Induced Pluripotent Stem Cell Repository (Reference Number GM25267).
Michael Longaker (NCL)	Marfan's syndrome	Model demonstrates osteogenic defect and TGFb dependent chondrogenesis and replicates phenotype of Marfan's hESC. Rescue observed with inhibition of TGFb signaling.
Amander Clark (NCL)	Chromosomal Aneuploidies	In aneuploid hESC lines, gene expression is active in the trisomic chromosome across different trisomies, resulting in altered development.

*Note:* List of notable, CIRM‐funded projects supporting the development and use of tools to explore pathological mechanisms and discover novel therapeutic approaches. PI, disease areas or technologies targeted, and key contributions to the stem cell field are summarized.

Abbreviations: ASD, autism spectrum disorder; ATM, ataxia telangiectasia mutated; HD, Huntington's disease; hESCs, human embryonic stem cells; hiPSCs, human induced pluripotent stem cells; hPSCs, human pluripotent stem cells; LQTS, long QT syndrome; MAPK, mitogen activated protein kinase; NCL, New Cell Lines initiative; PI, principal investigator; PI3K, phosphoinositide 3‐kinase; TGFb, transforming growth factor beta; TNT, Tools and Technology initiative.

Schizophrenia strikes in the prime of life with devastating impacts upon patients and their families. Medications have evolved little in decades, address only a subset of symptoms and can lead to serious and permanent side effects. Patients must also endure a trial and error period to identify an effective and tolerable regimen.^15^ Thus, treatment of schizophrenia represents a clear unmet medical need. Unfortunately, the etiology of schizophrenia remains poorly understood.^3^ To address this, Dr Fred Gage, with NCL grant support, developed human induced pluripotent stem cell (hiPSC) lines from five unaffected controls and four patients with a history of schizophrenia with varying genetic backgrounds. After differentiating the cells to neurons in vitro, genes previously linked to schizophrenia in genome‐wide association studies were confirmed to have altered gene expression in the patient samples, and new gene expression changes were also identified. Patient neurons showed fewer neurites and decreased connectivity, and these deficits were rescued by the antipsychotic drug loxapine. Taken together, these early observations suggest that hiPSC can be used to model a polygenic disorder and potentially to identify patient specific treatments. This line of inquiry is continuing to contribute to our understanding of disease.^16^, ^17^

## 3 NOVEL ASSAYS

### 3.1 In vitro pluripotency assay

The development of hiPSC and the explosion of new human pluripotent stem cell (hPSC) lines has given impetus to develop simpler and less expensive in vitro assays for pluripotency. The formation of teratomas after implantation in immune deficient mice has been the gold standard assay to assess pluripotency of hPSC lines^18^ and the teratoma risk of pluripotent stem cell derived therapies. However, teratoma assays are costly and require both animal use and expertise in pathology to interpret the results. To address this challenge, under her TNT award, Dr Jeanne Loring's laboratory developed an in vitro teratoma assay surrogate, the Pluritest.^19^ This user‐friendly bioinformatics tool can assess “stemness” in pluripotent stem cell lines. By comparing the transcriptome of a test cell line to the those from a large number of known pluripotent cell lines, the tool can distinguish pluripotent cells from teratocarcinoma cells, reprogramming cells, differentiating cells, and multiple differentiated cell types. Pluritest has been used by numerous groups and has been licensed by Coriell Institute for Medical Research (https://www.coriell.org/News/Read/HWmJCWaPqkCRtGcjRVQ45Q/coriell‐institute‐licenses‐pluritest‐‐a‐novel‐stem, https//pluritest.org/). Pluritest speeds the screening of new pluripotent cell lines, saving money, and reducing animal use.

### 3.2 In vitro cardiac toxicity assay

Arrhythmia liability is a leading cause of drug withdrawals, and the International Council for Harmonization of Technical Requirements for Pharmaceuticals for Human Use (ICH) guidelines recommend screening for inhibition of human ether‐a‐go‐go (hERG) gene product, a frequent arrhythmia driver, during the drug development process.^20^ Dr Patrick McDonough's team at Vala Sciences developed kinetic image cytometry (KIC), a multiparametric, mid‐throughput, microscopy‐based screening technique. KIC images calcium transients, the downstream consequences of action potentials, in living CMs, to elucidate potentially dangerous changes reflective of arrhythmia.^21^ Because of this utility, Vala's technology was incorporated in EPA's ToxCast screening project to assess potential risks of household and industrial chemicals (http://www.valasciences.com/news/epa‐awards‐contract‐to‐vala‐sciences‐to‐screen‐chemicals‐for‐human‐health‐i and https://cfpub.epa.gov/si/si_public_record_report.cfm?Lab=NCCT&dirEntryId=307716ad).^22^ This technology is commercially available. Vala's work is complemented by in vitro models of human congenital Long QT Syndromes (LQTS) 2 and 3 developed by Dr Bruce Conklin's laboratory at the Gladstone Institutes, with NCL support. These hiPSC‐CM models, derived from affected patient fibroblasts, display LQTS disease phenotypes as confirmed by electrophysiology and calcium transients.^4^

## 4 MANUFACTURING PROCESS TOOLS

### 4.1 Differentiation of microglia from pluripotent cells

Microglia, the immune cells resident in the brain, have long been known to play a role in the pathology of neurodegenerative brain disorders such as Alzheimer's disease (AD).^23^, ^24^ However, efforts to study human microglia were challenging, as they differ substantially from their counterparts in mice, and methods to derive them directly from human stem cells or progenitors were complex and inefficient. To address these challenges, Dr Matthew Blurton‐Jones' laboratory at University of California, Irvine (UCI)^25^, ^26^ developed a fully defined method to generate microglia from iPSCs. iPSC derived microglia like cells (iMGL) cluster with both fetal and adult microglia by gene expression. iMGL also display functional characteristics of microglia including cytokine responses, extending processes to explore their in vivo environment and phagocytosis of β‐amyloid peptide after transplantation into immune deficient AD mice. These cells have enabled study of microglia in AD pathogenesis both in vitro and in vivo in a hybrid human microglia, mouse brain model.^27^ This technology has since been licensed to Fujifilm Cellular Dynamics and marketed as iCell Microglia (https://fujifilmcdi.com/products-services/icell-products/microglia/).

### 4.2 Automated differentiation optimization

To take advantage of the potential of stem cells to give rise to specific desired cell fates, scientists typically develop differentiation protocols through an iterative trial and error process, experimenting with the timing and concentration of growth factor, cytokine, or small molecule addition to the cell culture in order to optimize yield and purity of the cells. Unfortunately, this process is slow, labor‐intensive, and expensive, due to the high cost of reagents. To address this bottleneck, an award to Dr Marc Unger of Fluidigm Corporation spearheaded the development of Callisto, an automated microfluidic‐based cell culture system that allows investigators to screen multiple different conditions using minimal quantities of reagents. Once programmed, Callisto can conduct automated growth factor additions and media changes for up to 3 weeks, reducing reagent and staff costs. This CIRM‐funded tool has been commercially available since 2015 (https://www.fluidigm.com/search?query=Callisto).

### 4.3 Genetic modification of stem cells

The ability to edit genomes in pluripotent stem cells is a powerful way to study the function of specific genes during or after differentiation to specific lineages, as well as to correct disease‐causing mutations for therapeutic purposes or to generate isogenic lines for in vitro disease modeling. Historically, gene editing of pluripotent stem cells has been challenging, due to low efficiencies of transduction of viral vectors carrying template DNA. To address this bottleneck, Dr David Schaffer's lab at UC Berkeley used an in vitro evolution strategy to generate and screen for novel adeno‐associated virus (AAV) capsids that could infect hPSCs with high efficiency. Consistent with earlier reports, delivery of template DNA via AAV vectors confers relatively high levels of homologous recombination, thus the improved transduction efficiencies made gene editing of pluripotent stem cells relatively straightforward.^28^ In addition to identifying AAVs that can selectively infect specific cell types, this method can also be used to identify mutant AAV capsids that circumvent the pre‐existing immunity to common AAV serotypes, making it particularly valuable for gene therapy. In fact, this CIRM funded technology formed the basis for 4D Molecular Therapeutics, a California gene therapy company that has attracted major pharmaceutical industry partners (including AstraZeneca, Roche, and Pfizer), and is poised to initiate clinical trials in 2020 using their directed evolution technology.

## 5 GOOD MANUFACTURING PRACTICES

Research manufacturing processes are often small scale, require extensive manual handling, and employ reagents designated for research only that are inappropriate for human use. Multiple CIRM awards have addressed these challenges.

### 5.1 Xeno‐free, scalable manufacturing of pluripotent cells

An award to Dr Larry Couture of City of Hope addressed two core manufacturing bottlenecks to the clinical use of hPSCs: the reliance on xenobiotic reagents such as mouse feeder cells and the mouse tumor‐derived extracellular matrix mixture, Matrigel, as well as the limited scale at which adherent hPSCs could be propagated. By taking advantage of Rho kinase inhibitors^29^ and survival signals from cell‐cell adhesion in hESCs,^30^ the City of Hope team was able to grow hESC as clusters in suspension, thereby replacing both feeder cells and Matrigel. The team identified a specific cellular cluster size that maintains hESC pluripotency, and because the cells are grown in suspension, the method is highly scalable to larger bioreactors and requires fewer manual manipulations. This suspension culture method is good manufacturing process (GMP) compliant, xenobiotic‐free and scalable.^31^ This method has been generalized to multiple hESC lines and cited 125 times as of this writing. The tool has been adopted by multiple groups, including two conducting CIRM affiliated IND‐enabling stage projects and three earlier stage preclinical programs (unpublished data).

Another challenge for pluripotent stem cell‐derived therapies is to induce differentiation to the desired therapeutic cell type using clinically compatible methods, first at the research scale and then at larger scale for clinical application. Two examples of successful efforts to differentiate therapeutically relevant cell types at high purity from pluripotent cells are described below.

### 5.2 Xeno‐free, scalable manufacturing of CMs

The ability to replace cardiac myocytes lost to a myocardial infarction has the potential to prevent progression to heart failure. At an approximate dose of 1e9 cardiac myocytes per patient, to be practical this strategy requires efficient, inexpensive cell production at a very large scale and high purity. Under his CIRM TNT award, Dr Joseph Gold at City of Hope replaced expensive growth factors with small molecules to develop a xenobiotic‐free, GMP compliant method to differentiate cardiac myocytes at high purity, making the generation of billions of myocytes per run both feasible, and, because the process uses bioreactors, scalable.^32^ The method uses the suspension hESC cultures developed by Dr Couture, above and has been cited 86 times as of this writing. Material made by this process is in use by two preclinical stage translational projects and will be employed in two future clinical trials currently conducting IND enabling studies (unpublished data).

## 6 TRANSLATIONAL TOOLS

Translational bottlenecks including limitations of established preclinical models, inefficient cell engraftment, and clinical delivery methods were also a focus of the Tools and Technology program.

### 6.1 Animal models of engraftment

Because they share structure and arrhythmia vulnerability with humans, large animal models are employed to assess efficacy and arrhythmia risk of cell transplants. Unfortunately, evaluating the risk of human cells in pigs, the workhorse large animal model for traditional drug cardiac safety evaluation, is infeasible. Even in the presence of immune suppression, robust xenobiotic rejection of human cells limits the duration of cell transplant studies. Rejection could itself pose an arrhythmia risk, complicating interpretation of results. Immune deficient pig models are still being established. A surrogate‐in‐surrogate strategy using pig iPSC‐CM was deemed infeasible, as the behavior of pig iPSC are not similar to human iPSC, rendering them a poor surrogate for human cells.^33^, ^34^

To address this gap, Dr Joseph Wu's team at Stanford sought to develop an alternative large animal model of PSC‐CM transplantation to evaluate the safety and efficacy of human CM transplantation. They generated non‐human primate (NHP) iPSC, differentiated them to cardiac myocytes, and then characterized them both in vitro and in vivo. The team published that NHP iPSC‐CMs are comparable to hiPSC‐CM in their ability to improve postinfarct cardiac function.^35^ Both cell types appear to act through similar mechanisms; they prevent remodeling and share transcriptional and metabolomic profiles in response to hypoxia. Therefore, these cells are an appropriate source for modeling the behavior of hiPSC‐CM in vivo. Furthermore, unlike in pigs, an immunosuppression regimen successfully retains human grafts in the NHP heart (Dr J. Wu, personal communication), perhaps because NHP are evolutionarily closer to humans. Therefore, this model enables the study of both allogeneic surrogate‐in‐surrogate transplants as well as transplants of the human cell candidate, such that the immune response can be decoupled from effects of the grafted cells and longer studies can be conducted. This model is currently being used in CIRM funded preclinical studies that will support a clinical trial (Dr Wu, unpublished data).

### 6.2 Cell delivery to human brain

Adequate and accurate delivery of therapeutic intervention to targeted sites is critical for both safety and efficacy of cell and gene therapies. However, covering a large brain region, such as the putamen, with traditional methods requires multiple injections with a straight cannula. Each brain penetration increases the bleeding risks to the patient, and the resultant delivery pattern is constrained to lines of single, parallel needle tracks. To address these limitations, Dr Daniel Lim's award funded the development of the radial branched deployment delivery device (RBD). The device employs a collet with a side port through which a switchable cannula is placed. After entry through a single site, the surgeon can rotate the angle of the side port to deliver cells through the cannula at multiple angles at a given depth. The depth of delivery can then be modified to achieve three‐dimensional coverage of a targeted brain region in a tree like pattern. The RBD can also be used with existing magnetic resonance imaging compatible frames to allow real time targeting. In this way, the surgeon can achieve tailored coverage of a brain target. This patented technology achieved a 510K filing and is available to investigators for further testing.^36^

### 6.3 Xenobiotic‐free matrices for cell delivery

Achieving the vision of regenerative cell therapy requires overcoming a combination of shear stress during delivery, a hostile in vivo disease environment, and anoikis that limit post‐transplantation survival.^37^ Without a formulation containing survival cues, more than 90% of cells can be lost post‐transplant, representing an enormous cost inefficiency of cell therapy that, if not addressed, will constrain commercial success. Pro‐survival formulations such as Matrigel provide growth factors and integrin ligands and can improve engraftment,^38^, ^39^ albeit at increased risk of adventitious agents. To reduce the risk presented by Matrigel, CIRM funded development of xenobiotic‐free formulations to promote transplanted cell survival. Under his TNT award, Dr David Schaffer's lab at UCB^40^ developed a xenobiotic‐free hydrogel to protect dopaminergic neurons from shear stress during delivery and anoikis (by providing integrin ligands and protecting cells from shear stress). The hydrogel improved dopaminergic neuron engraftment by fivefold in vivo. The stiffness of the material also promotes dopaminergic neuron maturation in vivo,^40^ resulting in more efficient use of cell product.

Dr Sarah Heilshorn's team at Stanford developed the xenobiotic‐free Shear‐thinning Hydrogel for Injectable Encapsulation and Long‐term Delivery (SHIELD) under her award. SHIELD can be tailored to individual cell types to protect them from shear induced cell death upon delivery. In vivo testing in an animal model of peripheral arterial disease demonstrated hiPSC derived endothelial cells delivered in SHIELD displayed up to 10‐fold better post‐transplant survival compared to cells in saline alone.^41^ These CIRM funded hydrogel systems are in use by multiple groups. If the improved engraftment seen in preclinical models translates to the clinic, these hydrogel systems could prove transformative for cell replacement strategies.

## 7 LESSONS AND OPPORTUNITIES

In this perspective, we have described examples (Table 2) of outcomes from a sampling of CIRM's New Cell Lines and Tools and Technologies investments. When these RFAs were first issued starting in 2007, the therapeutic potential for pluripotent stem cells was recognized, but the knowledge and capabilities for exploiting it was elementary. Federal restrictions limited use of hESCs, and iPSCs were a new discovery.^42^, ^43^ Unsurprisingly, the first awards supported through these programs developed fundamental tools for exploration, that is, those enabling the creation of hESC lines as a resource for study; new methods to culture, maintain, and manipulate human stem cells for therapeutic discovery, and novel protocols to genetically modify hESCs and differentiate them into desired cell types for therapeutic application. Tools to visualize and study stem cell behaviors in their natural or contrived environments were also an early priority.

**TABLE 2:** Featured awards from CIRM's tool focused initiatives

PI, program	Institution	Bottleneck	Technology
Couture (TNT)	COH	Manufacturing process	Xenobiotic‐free, scalable, suspension hESC manufacturing process
Gold (TNT)	COH	Manufacturing process	Xenobiotic‐free, small molecule hESC‐CM manufacturing process
Blurton‐Jones (TNT)	UCI	Manufacturing process tool	Defined microglia differentiation process
Unger (TNT)	Fluidigm	Manufacturing process tool	Callisto, an automated microfluidic instrument enabling multifactorial screening of cell culture conditions
Schaffer (TNT)	UCB	Genetic modification	Method to identify novel AAV capsids for more specific gene delivery and lower immunogenicity
Loring (TNT)	TSRI	Assays	PluriTest Assay for verifying pluripotency
McDonough (TNT)	Vala Sciences	Assays	Kinetic image cytometry, hPSC‐CM based, microscopic cardiotoxicity assay
Conklin (NCL, TNT)	UCSF, Gladstone	Human disease model	In vitro models of genetic long QT syndrome
Gage (NCL)	Salk	Human disease model	In vitro schizophrenia model
Wu (TNT)	Stanford	Animal model	NHP model for allogeneic and autologous PSC derived CM transplant studies
Lim (TNT)	UCSF	Cell delivery	Radial branched cell delivery device
Schaffer (TNT)	UCB	Cell delivery	Xenobiotic‐free, shear protecting hydrogel for in vivo cell delivery
Heilshorn (TNT)	Stanford	Cell delivery	Xenobiotic‐free, shear protecting hydrogel for in vivo cell delivery

*Note:* List of CIRM supported projects that are highlighted in this Perspective including PI (principal investigator), Program, grantee institution, the technical bottleneck addressed, and a description of the developed technology.

Abbreviations: AAV, adeno‐associated virus; COH, City of Hope; hESCs, human embryonic stem cells; hESC‐CMs, human embryonic stem cell‐derived cardiomyocytes; hPSC‐CM, human pluripotent stem cell‐derived cardiomyocytes; NCL, New Cell Lines initiative; NHP, nonhuman primate; PI, principal investigator; PSCs, pluripotent stem cells; TNT, Tools and Technologies initiative; TSRI, The Scripps Research Institute; UCB, University of California, Berkeley; UCI, University of California, Irvine; UCSF, University of California, San Francisco.

In subsequent funding cycles, the value of iPSCs had been clearly established, and thus efforts shifted toward developing more efficient and clinically acceptable ways to generate and genetically engineer iPSCs for opening new doors to autologous therapy. Gradually, as knowledge was gained and therapeutic candidates emerged, the most pressing technological needs shifted toward those enabling clinical translation to broad use, including the development of preclinical models for establishing disease‐modifying activity and safety of cell therapy products; tools and devices for delivering cell therapies to humans; GMP‐compatible methods for stem cell culture and differentiation; and technologies for controlling and tracking the fate of stem cells and their derivatives after transplantation. These trends illustrate the utility of a recurring funding opportunity focused on the most relevant technical hurdles of the day, which continually evolve and can be timely addressed through program updates.

Notably, many tools proposed to be cutting edge at the time have been subsequently replaced by newer discoveries, such as CRISPR/Cas9 for gene editing, and the development of efficient, nonintegrating methods for iPSC generation. This comes as no surprise, given the intense focus and keen interest in the rapidly advancing field of regenerative medicine, which is further accelerated by parallel developments the related disciplines of genomics platform development, materials engineering, and data science. However, even where new tools become obsolete, they represent necessary and fundamental steps upon which subsequent generations of tools can be discovered and built, gradually moving toward the optimal utility.

Furthermore, the use of new tools can reveal new technical challenges that were previously not appreciated. Interestingly, while some of the initial challenges were overcome quickly, there remain several technical bottlenecks that have always been appreciated as significant, but for which only incremental progress has been made over the past decade. These include a lack of methods to produce PSC derivatives in vitro with mature, functional phenotypes; fundamental gaps in knowledge about the biology of human cell types in their natural environment, and even the molecular and cellular composition of the tissues to be replaced or modified by a therapeutic stem cell technology.

Another persistent challenge has been the lack of clinically relevant animal models for testing human cell‐based therapeutics, and the related obstacle of immune barriers when testing and developing new treatments that are intended to engraft and survive long term. There is no doubt that continued tool discoveries around 3D tissue models, organoid culture, and the parallel advances in genomics analysis will begin to shed light on these problems. The ability to combine such efforts and apply multidisciplinary problem solving to longstanding hurdles has been an important outcome of CIRM's early initiatives, illustrating how a Tool‐focused approach can act as a nexus for stimulating new ideas and innovations to collectively move a field forward.

## 8 CONCLUSION

CIRM has funded the development of a wide array of tools to advance regenerative medicine from the discovery to clinical development stage. Through its TNT and NCL programs, CIRM engaged engineers, material scientists, process development engineers, and bioinformatics experts in the stem cell field who as of 2019, have contributed to over 300 scientific publications, 63 novel inventions and technologies, and 13 issued patents. Additional impacts have been realized through new marketed products and instruments, new technologies poised for clinical trials, and the launch of new for profit companies. These multidisciplinary investigators continue to work in the stem cell field and have collectively advanced regenerative medicine both by the tools they developed as well as the discoveries that were enabled by them. The ultimate impact of this CIRM investment will continue to unfold in the coming years.

## CONFLICT OF INTEREST

The authors declared no potential conflicts of interest.

## AUTHOR CONTRIBUTIONS

L.R.C.: conception and design, manuscript writing, final approval of manuscript; K.A.S.: conception and design.
